# Intracisternal delivery of NFκB-inducible scAAV2/9 reveals locoregional neuroinflammation induced by systemic kainic acid treatment

**DOI:** 10.3389/fnmol.2014.00092

**Published:** 2014-12-02

**Authors:** Olivier Bockstael, Liliane Tenenbaum, Deniz Dalkara, Catherine Melas, Olivier De Witte, Marc Levivier, Abdelwahed Chtarto

**Affiliations:** ^1^Laboratory of Experimental Neurosurgery and Multidisciplinary Research Institute, Institut de Recherche Interdisciplinaire en Biologie Humaine et Moléculaire – Université Libre de BruxellesBruxelles, Belgium; ^2^Neurosurgery Service and Laboratory of Cellular and Molecular Neurotherapies, Department of Clinical Neuroscience, Lausanne University HospitalLausanne, Switzerland; ^3^INSERM, U968Paris, France; ^4^Sorbonne Universités, UPMC Univ. Paris 06, UMR_S 968, Institut de la VisionParis, France; ^5^CNRS, UMR_7210Paris, France

**Keywords:** AAV, neuroinflammation, inducible vector, cisterna magna, cerebrospinal fluid

## Abstract

We have previously demonstrated disease-dependent gene delivery in the brain using an AAV vector responding to NFκB activation as a probe for inflammatory responses. This vector, injected focally in the parenchyma prior to a systemic kainic acid (KA) injection mediated inducible transgene expression in the hippocampus but not in the cerebellum, regions, respectively, known to be affected or not by the pathology. However, such a focal approach relies on previous knowledge of the model parameters and does not allow to predict the whole brain response to the disease. Global brain gene delivery would allow to predict the regional distribution of the pathology as well as to deliver therapeutic factors in all affected brain regions. We show that self-complementary AAV2/9 (scAAV2/9) delivery in the adult rat cisterna magna allows a widespread but not homogenous transduction of the brain. Indeed, superficial regions, i.e., cortex, hippocampus, and cerebellum were more efficiently transduced than deeper regions, such as striatum, and substantia nigra. These data suggest that viral particles penetration from the cerebrospinal fluid (CSF) into the brain is a limiting factor. Interestingly, AAV2/9-2YF a rationally designed capsid mutant (affecting surface tyrosines) increased gene transfer efficiency approximately fivefold. Neurons, astrocytes, and oligodendrocytes, but not microglia, were transduced in varying proportions depending on the brain region and the type of capsid. Finally, after a single intracisternal injection of scAAV2/9-2YF using the NFκB-inducible promoter, KA treatment induced transgene expression in the hippocampus and cortex but not in the cerebellum, corresponding to the expression of the CD11b marker of microglial activation. These data support the use of disease-inducible vectors administered in the cisterna magna as a tool to characterize the brain pathology in systemic drug-induced or transgenic disease models. However, further improvements are required to enhance viral particles penetration into the brain.

## INTRODUCTION

Increasing evidence suggests that global brain neuroinflammation is a key factor in the progression of neurodegenerative diseases (Dexter and Jenner, 2013). However, a controlled inflammatory response can also be beneficial for brain repair (London et al., 2013). Therefore, approaches aiming at the reduction of neuroinflammation should use conditional expression of the interfering factor in excessive stress conditions (Brown and Neher, 2014). A reduction of toxic intracerebral conditions such as neuroinflammation could possibly be achieved through global brain gene delivery while avoiding the risk of surgery-related local inflammatory reactions.

Intraperitoneal injection of kainic acid (KA), a model of temporal lobe epilepsy (Williams et al., 2009), is known to induce a locoregional inflammatory response in the hippocampus and in the cortex (Somera-Molina et al., 2009) but not in the cerebellum (Rong and Baudry, 1996).

In order to specifically express disease-interfering genes it is desirable to use a disease-regulated vector providing a similar pattern of transgene expression.

Gene delivery to the whole brain can be achieved through intravenous administration followed by *trans*-blood–brain barrier (BBB) passage (Duque et al., 2009; Foust et al., 2009; Mccarty et al., 2009; Fu et al., 2011; Gray et al., 2011) or by intra-cerebro-spinal fluid (CSF) administration followed by passage through the CSF-brain barrier (Fu et al., 2003, 2010; Samaranch et al., 2012, 2013; Gray et al., 2013). The BBB and CSF-brain barriers being of different nature, the regions and cell types transduced as well as the immune reaction toward the viral capsids and transgene products are expected to be different. Indeed, intracisternal injections are characterized by a reduced immune response and maintenance of transgene expression despite pre-existing immunity as compared to intravenous injections. However, Samaranch and collaborators (Samaranch et al., 2012) showed that, at least in monkeys, pre-existing anti-AAV antibodies abrogated brain transduction following delivery into the cisterna magna CSF. Another advantage of intracisternal versus intravenous injections is the lower biodistribution in peripheral organs (Gray et al., 2013).

Intracisternal injections of self-complementary (sc) AAV2 in juvenile mice combined with systemic mannitol administration (Fu et al., 2003, 2010), of scAAV2/9 and scAAV2/7 in non-human primates (Mccarty et al., 2009; Gray et al., 2011, 2013; Samaranch et al., 2013) as well as of scAAV2/9 and scAAV2/1 in the cat (Bucher et al., 2013) have been described with reports of neurons and astrocytes being transduced in the brain and spinal cord.

Largely documented models hypothesize that the CSF produced in the choroid plexus of the lateral ventricles, travels to the cisterna magna, then to the subarachnoid space and further moves into the brain in the perivascular Virchow-Robin space surrounding penetrating cerebral arteries. Consistently with this model, large molecules tracers (2000 kD) injected in the cisterna magna accumulate along perivascular spaces mainly in superficial layers (cortex; Iliff et al., 2012). As predicted by these models, AAV-mediated gene delivery after intracisternal injection was maximal in regions close to Virchow-Robin subarachnoid space (cerebellum and cortex) and reduced in deeper regions (Samaranch et al., 2013).

It has recently been shown that gene delivery to the retina and the brain of neonatal mice through systemic intravenous injection of AAV2/9 was enhanced by a capsid mutant in which two tyrosine residues presumably involved in capsid ubiquitination were replaced by phenylalanines (Dalkara et al., 2012). Here we investigated whether such mutations could also enhance gene delivery to the brain after intra-CSF delivery.

We compared the distribution and cell-type specificity of gene transfer into the brain after intracisternal injection of scAAV2/9 and the 2YF capsid mutant in the adult rat brain. With both vectors, gene transfer was prominent in the cerebellum and in the cortex with neurons, astrocytes and oligodendrocytes, but not microglia, being targeted in different proportions. The 2YF capsid mutant affected both the global efficiency and the proportions of neurons and glial cells.

We showed that, as expected, intraperitoneal KA injection induced transgene expression from a cisternally injected NFκB-inducible scAAV2/9-2YF vector in the cortex and hippocampus but not in the cerebellum.

Pathology-inducible gene transfer could be used to probe pathological events as well as for regulated therapeutic gene delivery specifically in diseased brain regions.

## MATERIALS AND METHODS

### PLASMIDS

The pSC-NF8-d1-EGFP plasmid containing eight NFκB responsive elements (two blocks of four consensus sequences, separated by 16 bps) fused to a minimal CMV promoter has been previously described (Chtarto et al., 2013).

The pHpaI-EGFP self-complementary AAV (scAAV) vector was a kind gift from D. McCarty and R. J. Samulski (Mccarty et al., 2003).

The pAAV2/9 and pAd Delta F6 were a kind gift from the Pen Vector Core (University of Pennsylvania). pAAV2/9-2YF was previously described (Dalkara et al., 2012).

### VIRAL PRODUCTION

To produce recombinant scAAV2/9-CMV-EGFP, scAAV2/9-2YF-CMV-EGFP and scAAV2/9-NF8-d1-EGFP viral stocks, HEK-293T cells (5.0 × 10^6^ cells seeded on 10 cm plates) were co-transfected using polyethylenimine (PEI) from Polysciences, using a 5:1 (v:w) PEI:DNA ratio and a 2:3:5 molar ratio of vector plasmid, pAd plasmid and pAAV2/9 or pAAV2/9 2YF packaging plasmid expressing the AAV viral genes (rep gene from AAV serotype 2 and cap gene from AAV serotype 9 or from the 2YF tyrosine mutant). Fifty hours post-transfection, the medium was discarded and the cells were harvested by low-speed centrifugation and resuspended in Tris pH 8.0, NaCl 0.1 M. After 5 cycles of freezing/thawing, the lysate was clarified by 30 min centrifugation at 10 000 *g*, treated with benzonase (50 μ/ml) at 37°C for 30 min, and centrifuged at 10 000 *g* for 30 min to eliminate the residual debris. The virus was further purified by iodixanol gradient. Viral genomes (vg) were titrated by quantitative PCR using primers located in the SV40polyA sequence (forward primer: AGC AAT AGC ATC ACA AAT TTC ACA A; reverse primer: CCA GAC ATG ATA AGA TAC ATT GAT GAG TT; internal probe: 6FAM- AGC ATT TTT TTC ACT GCA TTC TAG TTG TGG TTT GTC-TAMRA). The PCR reaction included a pre-denaturing step of 10 min at 95°C followed by 40 cycles of the 2 following steps: 95°C for 15 s and 60°C for 1 min. (Lock et al., 2010). Titers were 1,4 × 10^13^ vg/ml, 2.0 × 10^13^ vg/ml and 5,8 × 10^12^ vg/ml, respectively, for rAAV2/9-CMV-EGFP, rAAV2/9-2YF-CMV-EGFP, and rAAV2/9-2YF-NF8-d1-EGFP.

### ANIMALS

For the analysis of constitutive transgene expression from the CMV promoter, adult female Wistar rats weighing approximately 200 g were used. For experiments with the KA model, adult male Sprague–Dawley rats weighing approximately 200 g were used. Animals were housed and treated according to the Belgian law. The protocols were in accordance with national rules on animal experiments and were approved by the Ethics Committee of the Faculty of Medicine of the “Université Libre de Bruxelles.”

Animals were anesthetized with a mixture of ketamine (Imalgène 1000, Merial; 100 mg/kg) and xylazin (Rompun, Bayer; 10 mg/kg) and placed on a Kopf stereotaxic frame (Kopf Instruments, Tujunga, CA, USA).

#### Intracisternal injections

The dura mater of the cisterna magna was made accessible by incision of the neck skin and gentle separation of the subcutaneous tissue and muscles under anesthesia as previously described (Liu and Duff, 2008). Ten minutes before virus injection a 25% solution of D-mannitol (Sigma–Aldrich, M8429-100G) diluted in water and sterilized by filtration was injected intraperitoneally (1 g/kg). Next, 10 μl of viral suspension diluted at 10^12^ vg/ml was slowly injected under microscope using a silicone-coated glass capillary connected to a 50 μl Hamilton syringe via polyethylene tubing. After viral infusion, the capillary was maintained in place for 2 min and then gently removed. The muscles were re-aligned, and the skin was sutured.

#### Intraperitoneal KA injections

Five weeks after the virus injection, induction of status epilepticus was performed as described earlier (Vermoesen et al., 2010). Briefly, consecutive intraperitoneal KA injections (5 mg/kg, diluted in PBS, Nanocs^®^) were administered at 30 min interval. If a rat was nearing status epilepticus, half-doses (2.5 mg/kg) were given in order to reduce mortality. Control rats were injected with saline (NaCl 0.9%).

#### Perfusions

Animals were sacrificed one week after intraperitoneal KA injection and perfused intracardially first with saline, then with 4% paraformaldehyde (PF4). Brains were post-fixed for 24 h in PF4.

### IMMUNOHISTOCHEMISTRY

For GFP staining, vibratome coronal brain sections (50 μm) were immunostained using anti-GFP antibodies using peroxidase staining as previously described (Chtarto et al., 2013).

For CD11b staining, the procedure was the same except that mouse monoclonal anti-CD11b (1:500, Serotec, MorphoSys, Dusseldorf, Germany) was used as a primary antibody and goat anti-mouse IgG conjugated with HRP (Molecular Probes, Invitrogen, Carlsbad, CA, USA, from TSA kit) as a secondary antibody.

Densitometric analysis of the staining was performed using the image J software (NIH, USA).

### QUANTIFICATION METHOD

Global brain transduction was quantified by two ways: cell density and staining proportion.

Cell density was assessed by counting cells on microscope pictures of known surface. All animals were photographed bilaterally on the same sections (same antero-posteriour level) at the same location (dorsal or lateral) under the same conditions (exposure, illumination) using an axioplan 2 microscope (Zeiss) with a 10X/0,30 plan NEOFLUAR objective (Zeiss). For cortex counting, 10 fields were photographed on five sections encompassing the cortex from frontal cortex to the beginning of the hippocampus. For hippocampus quantification, eight fields on four sections were photographed and counted.

Staining surface quantification was quantified on whole section mosaic pictures. One section out of six of all injected animals was DAB stained for GFP expression. All stained sections were photographed using the mosaic function of the Axiovision 4.8 software (Zeiss) on an imager M1 microscope (Zeiss) equipped with a 10X/0,3 EC plan NEOFLUAR objective (Zeiss). Section surface and stained surface were quantified in pixel using the threshold function of ImageJ software (NIH).

### IMMUNOFLUORESCENCE

#### GFP labeling

Coronal brain sections (50 μm) obtained using a vibratome (Leica Microsystems, Wetzlar, Germany) were incubated with a polyclonal rabbit anti-GFP (1:3000, Molecular Probes, Invitrogen, Carlsbad, CA, USA) followed by a biotin-streptavidin-cyanine 2 fluorescent labeling as previously described (Bockstael et al., 2012).

#### GFP: NeuN and GFP:GFAP co-labelings

For double immunofluorescence, the GFP labeling was combined with mouse monoclonal antibodies [anti-NeuN or anti-glial fibrillary acid protein (GFAP)] followed by donkey anti-mouse IgG coupled to cyanine 3 as previously described (Bockstael et al., 2012).

#### GFP: IbaI co-labeling

For GFP:IbaI double immunofluorescence, the above described GFP labeling was combined with goat anti-IbaI followed by a donkey anti-goat A.568 antibody as previously described (Bockstael et al., 2012).

#### GFP: Olig2 co-labeling

For GFP:Olig2 double immunofluorescence, a chicken monoclonal anti-GFP antibody and rabbit polyclonal anti-Olig2 IgG were used as previously described (Bockstael et al., 2012).

In order to better visualize the structure of the tissue, cerebellar sections were incubated in the Hoescht 33258 dye (Sigma–Aldrich) diluted at 1 μg/ml in TBS for 30 min.

### CONFOCAL MICROSCOPY

Co-labeling analysis was performed on pictures taken on at least three different sections using a LSM510 NLO multiphoton confocal microscope fitted on an Axiovert M200 inverted microscope equipped with C-Apochromat 40×/1.2 N.A. and 63×/1.2 N.A. water immersion objectives (Zeiss, Iena, Germany).

The 488 nm excitation wavelength of the Argon/2 laser, a main dichroic HFT 488 and a band-pass emission filter (BP500-550 nm) were used for selective detection of the green fluorochrome (Cy2, Alexa 488).

The 543 nm excitation wavelength of the HeNe1 laser, a main dichroic HFT 488/543/633 and a long-pass emission filter (BP565-615 nm) were used for selective detection of the red fluorochrome (Cy3).

Optical sections, two microns thick, 512 × 512 pixels, were collected sequentially for each fluorochrome. Z-stacks with a focus step of one micron were collected.

The data-sets generated were merged and displayed with the Zen software (Zeiss, 2009) and exported in LSM image format.

Counting was performed with the ImageJ 1.46a software (NIH, USA). Figures were prepared with Adobe Photoshop CS3 soft ware.

### STATISTICAL ANALYSIS

All the statistical analysis was performed using the GraphPad Software. Results were expressed as mean ± SD and statistical significance was evaluated with student *t*-test (to compare pairs of data sets). Differences were considered as significant when *p* < 0.05.

## RESULTS

### GLOBAL BRAIN TRANSDUCTION AFTER INTRACISTERNAL DELIVERY OF scAAV2/9 IS ENHANCED BY A RATIONALLY DESIGNED SURFACE TYROSINE CAPSID MUTATION

**FIGURE 1:**
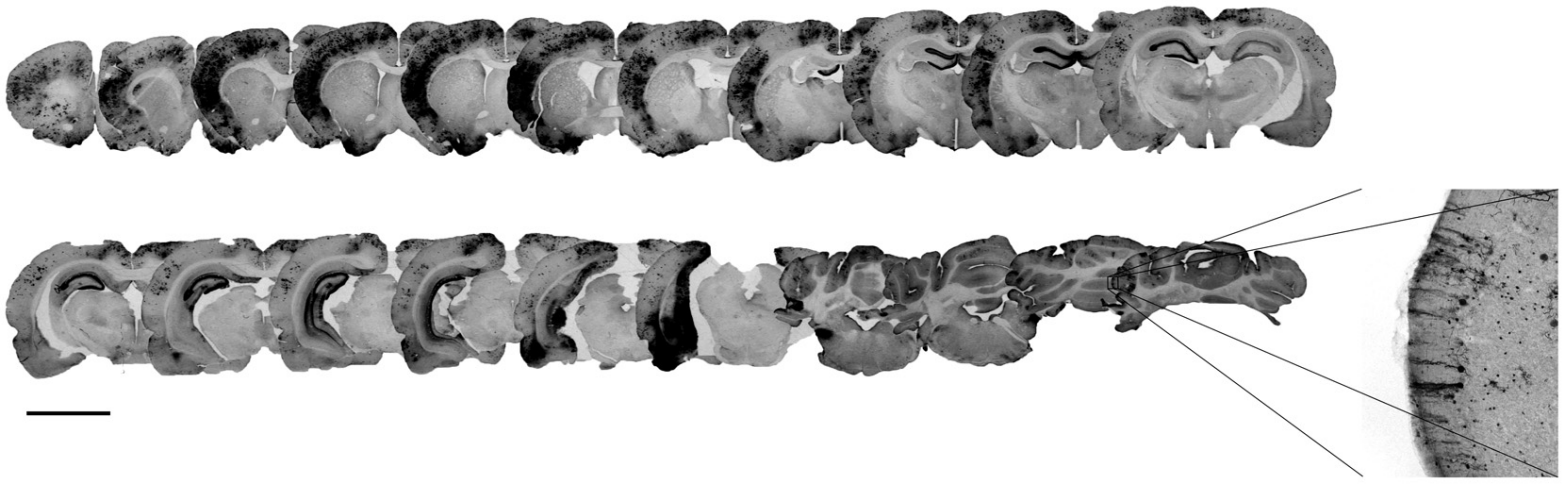
**Locoregional brain transduction after intracisternal delivery of scAAV2/9-2YF-CMV-EGFP.** The scAAV2/9-2YF-CMV-EGFP vector (10^10^ vg) was injected intracisternally. Five weeks after injection the rats (*n* = 5) were perfused and brain sections of 50 μm were labeled with anti GFP antibodies. The picture corresponds to an antero-posterior succession of coronal rat brain sections. The mean volume of transduction is 8.14% ± 3,3% of the whole brain. Scale bar: 5 mm.

**FIGURE 2:**
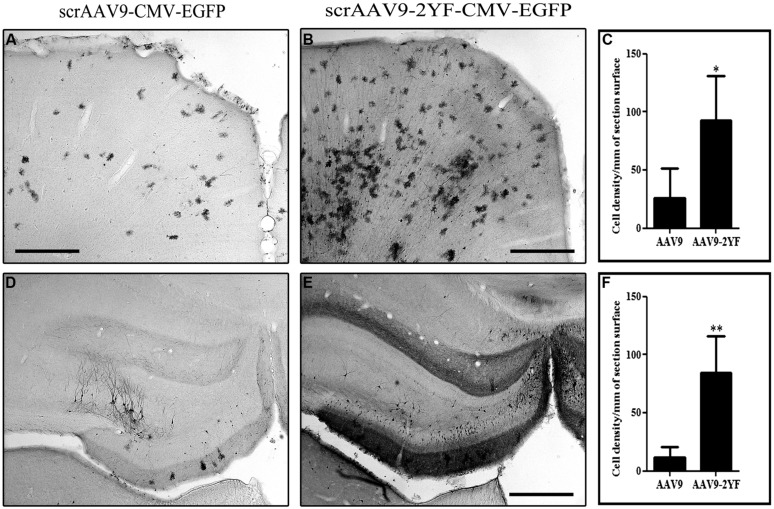
**Brain transduction after intracisternal delivery of scAAV2/9 is enhanced by mutation of two surface tyrosines.** Recombinant scAAV2/9-CMV-EGFP and scAAV2/9-2YF-CMV-EGFP (10^10^ vg) were injected intracisternally. Five weeks post-injection the rats were perfused and vibratome brain sections of 50 μm were labeled with an anti GFP antibody. The pictures show representatives examples of GFP-labeling in the cortex **(A**,**B)** and of the hippocampus **(D**,**E)**. Scale bar: 500 μm. The cell density in the cortex **(C)** and in the hippocampus **(F)** was evaluated as described in Section “Materials and Methods” and expressed as number of cells per square millimeter (mm^2^). Data are expressed as mean ± SD. The results obtained show more GFP-marked cells/mm^2^ of section surface in scAAV2/9-2YF compared to scAAV2/9 injected rats both in the cortex (**C**; *p* = 0,0395, student *t*-test) and in the hippocampus (**F**; *p* = 0,0063, student *t*-test).

It has been shown that gene delivery to the retina and the brain of neonatal mice as well as newborn macaques through systemic intravenous injection of AAV2/9 was enhanced by capsid tyrosine-to-phenylalanine mutant (Dalkara et al., 2012; Dehay et al., 2012) in a site presumably involved in the ubiquitination and the proteasomal degradation of AAV capsids (Zhong et al., 2008). We injected titer-matched scAAV2/9-CMV-EGFP and scAAV2/9-2YF-CMV-EGFP viral suspensions (10^12^ vg/ml) in the cisterna magna of adult female Wistar rats (scAAV2/9, *n* = 5; scAAV2/9-2YF, *n* = 5). One month post-injection, animals were perfused and brains processed for immunohistochemistry. **Figure 1** shows the antero-posterior distribution of GFP transgene expression in animals injected with the scAAV2/9-2YF-CMV-EGFP vector. The labeling was present on all coronal sections from the anterior cortex to the cerebellum. The right inset is a magnification of the cerebellar area, showing the typical morphological organization of Purkinje neurons. Interestingly, the density of GFP-positive cells was highest in the regions bordered by the Virchow-Robin subarachnoid space (cortex and cerebellum) and the third ventricle (hippocampus) in which the CSF from the cisterna magna circulates. **Figure 2** shows that the density of GFP-positive cells in the cortex (**Figures 2A–C**) and in the hippocampus (**Figures 2D–F**) was approximatively fivefold higher after scAAV2/9-2YF-CMV-EGFP (**Figures 2B,E**) injection as compared to scAAV2/9-CMV-EGFP (**Figures 2A,D**).

### INTRACISTERNAL DELIVERY OF scAAV2/9 AND scAAV2/9-2YF USING THE CONSTITUTIVE CMV PROMOTER MEDIATES GENE TRANSFER INTO NEURONS, ASTROCYTES AND OLIGODENDROCYTES

**FIGURE 3:**
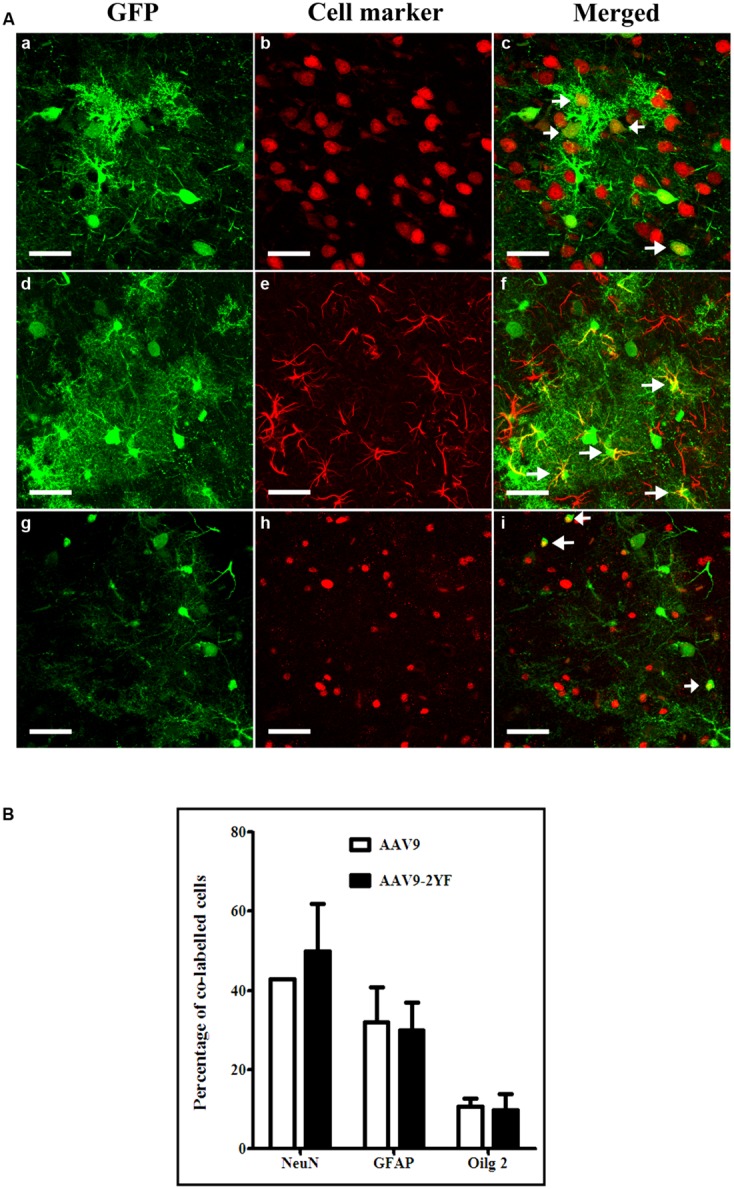
**Cellular specificity of scAAV2/9-CMV-EGFP and scAAV2/9-2YF-CMV-EGFP in the cortex. (A)** Five weeks after injection of scAAV2/9-CMV-EGFP (*n* = 5) or scAAV2/9-2YF-CMV-EGFP vector (*n* = 5) into the cisterna magna, brain sections were double-labeled with GFP (green fluorescence) and NeuN, GFAP or Olig2, (red fluorescence). Confocal pictures show GFP and cell-specific markers in double-labeled cells (yellow). GFP/NeuN, (a–c), GFP/GFAP, (d–f) and GFP/Olig2, (g–i). Arrowheads indicate double-labeled cells. Scale bar: 40 μm. **(B)** the proportions of co-labeled cells were not significantly different for the two vectors for NeuN (*p* = 0,3582, student *t*-test), GFAP (*p* = 0,7971, student *t*-test)) and Olig2 (*p* = 0,7622, student *t*-test). Open columns, scAAV2/9-CMV-EGFP; solid columns, scAAV2/9-2YF-CMV-EGFP. Data are expressed as means ± SD. Analysis was performed using confocal microscopy and counting the number co-labeled cells on five sections per animal (*n* = 3 rats for GFP/NeuN, GFP/GFAP and GFP/Olig2).

**FIGURE 4:**
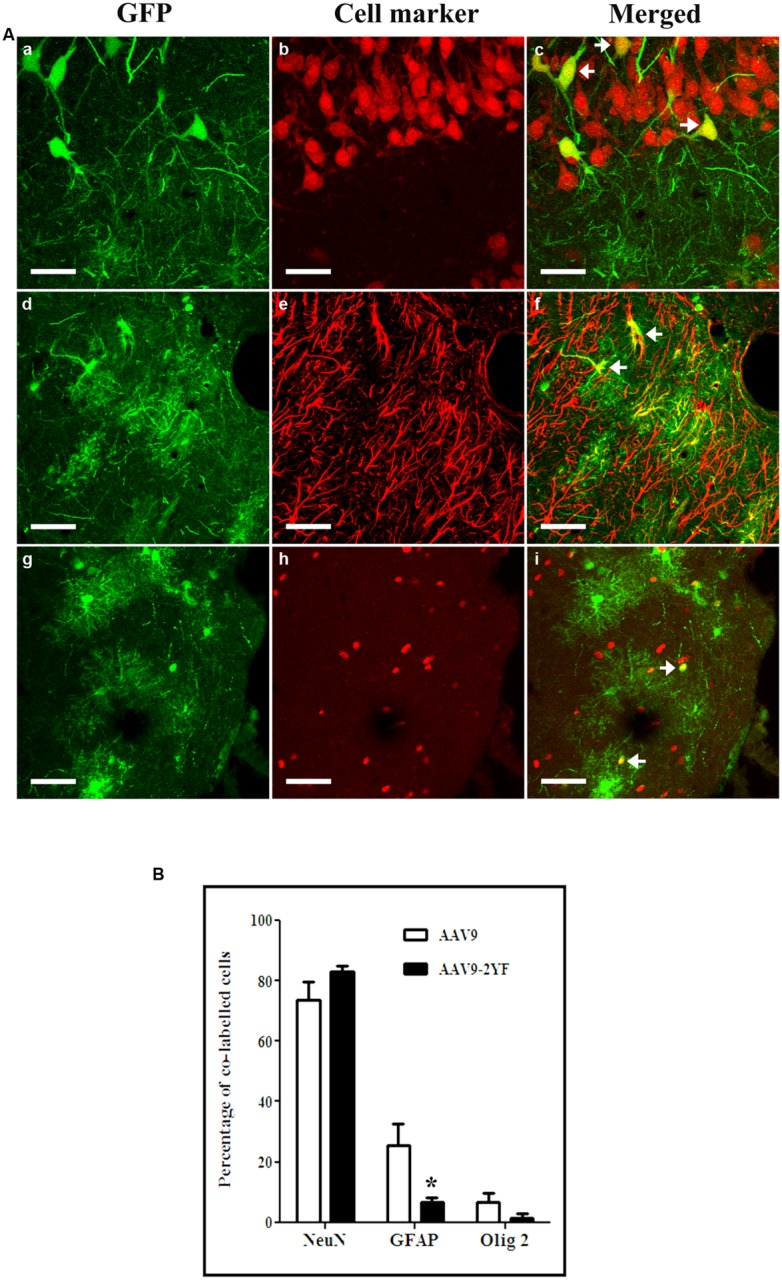
**Cellular specificity of scAAV2/9-CMV-EGFP and scAAV2/9-2YF-CMV-EGFP in the hippocampus. (A)** Five weeks after injection of scAAV2/9-CMV-EGFP (*n* = 5) or scAAV2/9-2YF-CMV-EGFP vector (*n* = 5) into the cisterna magna, brain sections were double-labeled with GFP (green fluorescence) and NeuN, GFAP, or Olig2 (red fluorescence). Confocal pictures show GFP and cell-specific markers in double-labeled cells (yellow). GFP/NeuN, (a–c), GFP/GFAP, (d–f) and GFP/Olig2, (g–i). Arrowheads indicate double-labeled cells. Scale bar: 40 μm. **(B)** the proportions of co-labeled cells were not significantly different for the two vectors for NeuN (*p* = 0,0602, student *t*-test) and Olig2 (*p* = 0,0500, student *t*-test), but were significantly different for GFAP (*p* = 0,0100, student *t*-test). Open columns, scAAV2/9-CMV-EGFP; solid columns, scAAV2/9-2YF-CMV-EGFP. Data are expressed as means ± SD. Analysis was performed using confocal microscopy and counting the number co-labeled cells on five sections per animal (*n* = 3 rats for GFP/NeuN, GFP/GFAP, and GFP/Olig2).

With both vectors, the majority of cells expressing GFP were neurons (labeled with NeuN marker; respectively, 42.9 ± 0.0% and 49.9 ± 11.8% for scAAV2/9 and scAAV2/9-2YF, in the cortex (**Figure 3**) and 73.7 ± 6% and 83.2 ± 1.8 in the hippocampus (**Figure 4**). Astrocytes and oligodendrocytes were also transduced with both vectors. Interestingly, the 2YF mutation differentially affected the proportion of GFP-positive cells expressing the GFAP astrocytic marker in the hippocampus (25.6 ± 6.9% of the GFP-positive cells for scAAV2/9 versus 6.8 ± 1.5% for scAAV2/9-2YF; *p* = 0.01; **Figure 4**) but not in the cortex (31.9 ± 9.4% of the GFP-positive cells for scAAV2/9 versus 30.0 ± 6.9% for scAAV2/9-2YF; *p* = 0.7971; **Figure 3**).

In contrast, the proportions of GFP-expressing cells which were Olig2-positive oligodendrocytes were not significantly different between both vectors, in both regions (Student *t*-test). However, a tendency for a lower proportion of GFP:Olig2 double labeled cells with the scAAV2/9-2YF mutant was observed in the hippocampus (*p* = 0.050; student *t*-test).

No GFP-positive cells expressed the IbaI marker of microglial cells with both vectors in both regions (data not shown).

### INFLAMMATION-INDUCIBLE GENE DELIVERY RESPONSE TO SYSTEMIC KA ADMINISTRATION IN THE CORTEX AND THE HIPPOCAMPUS

Injection of scAAV2/1-NF8-d1-EGFP into the hippocampal layers followed, 1 month later by intraperitoneal KA injection resulted in a strong transgene expression (Chtarto et al., 2013). However, in order to determine which regions of the brain are affected by the pathology, measured by NFκB activation, a global delivery of the vector is desirable.

**FIGURE 5:**
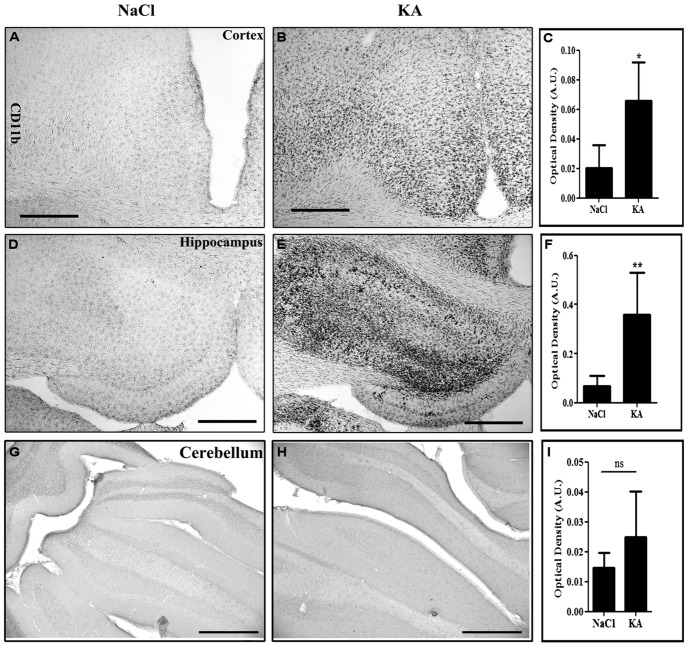
**Kainic acid (KA)-induced microglial activation evidenced by CD11b expression.** Vibratome brain sections (50 μm) were immuno-labeled using mouse anti-CD11b followed by streptavidin-biotin-peroxidase staining. The intensity of the staining of four random areas within the cortex **(A–C)**, hippocampus **(D–F)**, and the cerebellum **(G–I)**, on every section was quantified using the Image J program. Data are expressed as the mean optical density ± SD (*n* = 4 for each group of animals). The difference between KA and saline-treated groups was significant in the cortex (*p* = 0,0118, student *t*-test), in the hippocampus (*p* = 0,0084, student *t*-test) but not in the cerebellum (*p* = 0,5930, student *t*-test). Scale bar: 500 μm.

In the present study, we have therefore injected scAAV2/9-2YF-NF8-d1-EGFP (10^10^ viral particles in 10 μl) in the cisterna magna of adult rats. Two weeks later, intraperitoneal injections of KA (*n* = 8) or saline as a control (*n* = 6) were performed. Animals were perfused 2 weeks post-treatment. KA induced a strong inflammatory response characterized by prominent microglial activation as evidenced by CD11b labeling in the hippocampus and cortex but not in the cerebellum (**Figure 5**).

**FIGURE 6:**
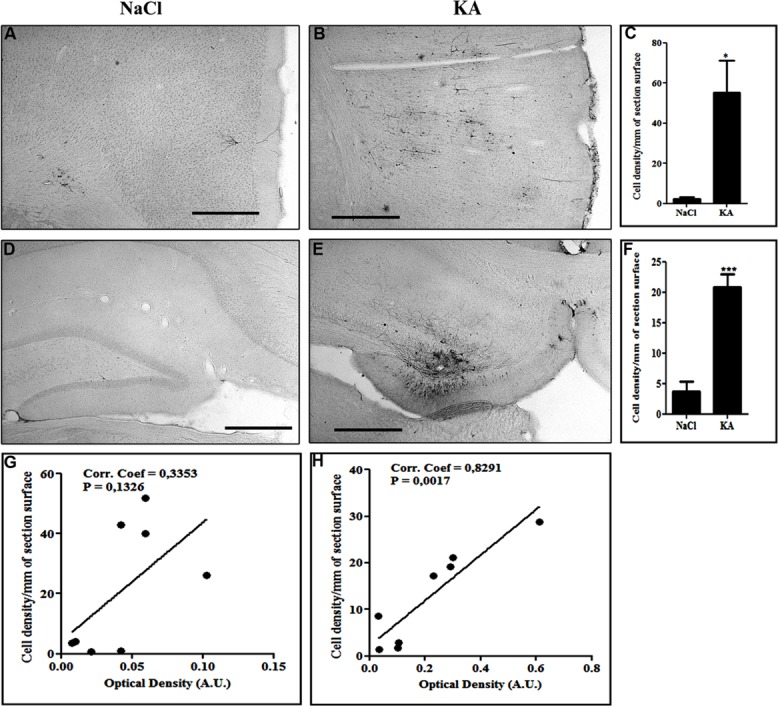
**Kainic acid induces NFκB activation-dependent transgene expression.** Recombinant scAAV2/9-2YF-NF8-d1-EGFP (10^10^ vg) was injected in the cisterna magna of Sprague–Dawley rats. The animals were kept in two groups: one group was intraperitoneally injected with KA (*n* = 8) 1 month post virus injection and the other group received only saline (*n* = 6). The animals were sacrificed two weeks after injection. Vibratome brain sections (50 μm) were immuno-labeled using anti-GFP antibody. The pictures show representatives examples of GFP-labeling in the cortex **(A,B)** and of the hippocampus **(D,E)**. The cell density in the cortex **(C)** and in the hippocampus **(F)** was evaluated as described in Section “Materials and Methods.” Data are expressed as mean ± SD. The results obtained in the cortex show a significant induction of GFP expression in the KA-treated rats compared to saline treated rats (*p* = 0,0207, student *t*-test). In the hippocampus the cell density in the KA-treated rats is highly increased compared to the saline-treated rats (*p* = 0,0005, student *t*-test). KA, kainic acid; saline, 0.9% NaCl. The cell density per mm^2^ of section surface was correlated to the CD11B intensity staining, respectively, in cortex (**G**; Correlation Coefficient = 0,3353 and P = 0,1326) and hippocampus (**H**; Correlation Coefficient = 0,8291 and *p* = 0,0017). Scale bar: 500 μm.

**FIGURE 7:**
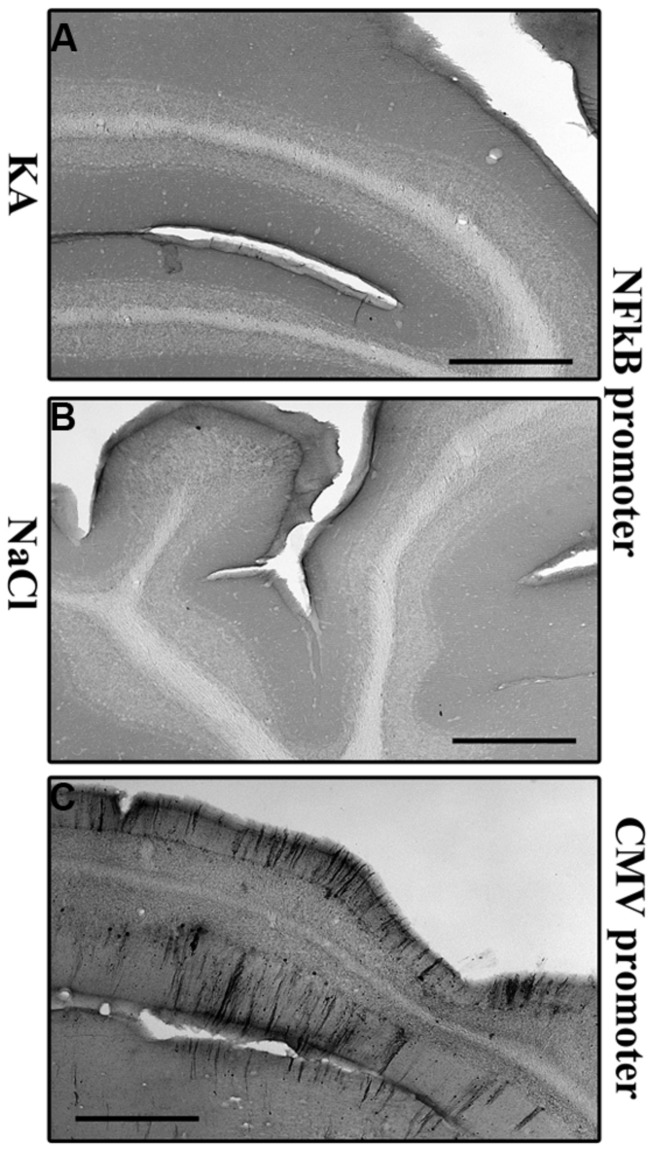
**Kainic acid does not induce NFκB activation-mediated transgene expression in the cerebellum.** KA (5 mg/kg) was injected intraperitoneally 5 weeks after intracisternal injection of scAAV2/9-2YF-NF8-d1-EGFP. Fifty μm coronal sections were labeled with GFP antibodies. Scale bar: 500 μm. The GFP staining is almost inexistent in KA- and saline-treated rats injected with scAAV2/9-2YF-NF8-d1-EGFP **(A,B)** while a strong GFP staining was demonstrated in rats injected with scAAV2/9-2YF-CMV-EGFP **(C)**.

We then wanted to analyze whether the regional pattern of GFP transgene expression driven by the NFκB-inducible promoter corresponded to microglial activation. Indeed, **Figure 6** shows representatives examples of GFP-labeling in the cortex (**Figures 6A,B**) and of the hippocampus (**Figures 6F**). The cell density in the cortex (**Figure 6C**) and in the hippocampus (**Figure 6F**) was evaluated. The results obtained in the cortex show a significant induction of GFP expression in the KA-treated rats compared to saline treated rats (*p* = 0.0207, student *t*-test). In the hippocampus the cell density in the KA-treated rats is highly increased compared to the saline-treated rats (*p* = 0.0005, student *t*-test). In contrast, in the untreated group, very few GFP-positive cells were evidenced (see **Figures 6A,D**). The intensity of the labeling in treated animals was variable both in terms of surface area and number of cells labeled. This variability could due to variability in the KA treatment efficacy (previously reported) or to variability in the injections, also observed in the AAVCMV group (see **Figure 2**). The cell density correlates to the intensity of the CD11b staining in the hippocampus (H; Correlation Coefficient = 0.8291 and *p* = 0.0017) but not in the cortex (G; Correlation Coefficient = 0.3353 and *P* = 0.1326). In addition, no GFP positive cell was evidenced in the cerebellum after scAAV2/9-2YF-NF8-d1-EGFP in the cisterna magna (**Figure 7**).

**FIGURE 8:**
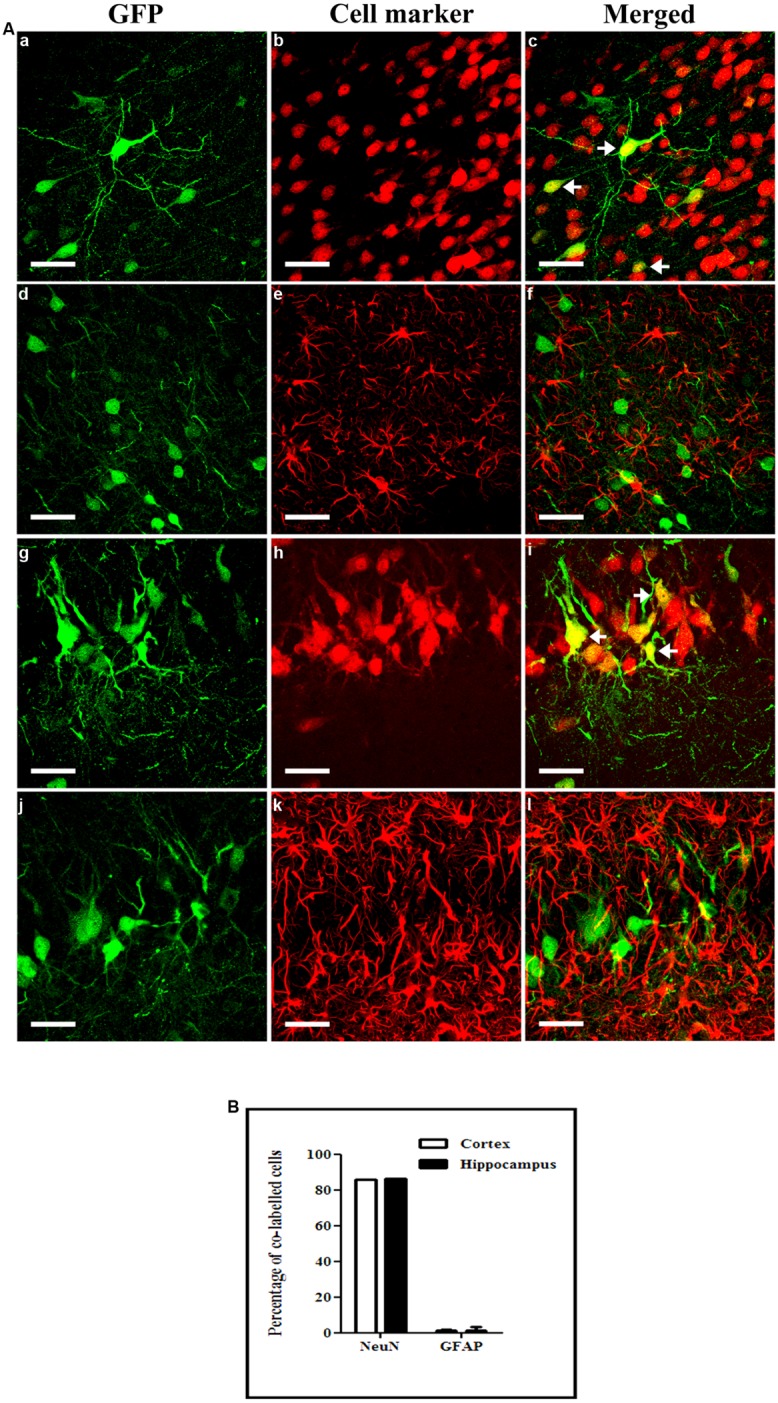
**Cellular specificity of NFκB activation-mediated transgene expression.** KA (5 mg/kg) was injected intraperitoneally 1 month after intracisternal injection of scAAV2/9-2YF-NF8-d1-EGFP. Fifty μm coronal sections were double-labeled with GFP (green fluorescence) and NeuN (GFP/NeuN, a–c in the cortex and g–i in the hippocampus) or GFAP (GFP/GFAP, d–f in the cortex and j–l in the hippocampus). Confocal pictures show GFP and cell-specific markers double-labeled cells (yellow; **A**). Arrowheads indicate double-labeled cells. Scale bar: 40 μm. The mean percentage of GFP-positive cells co-labeling with the GFAP and NeuN markers are shown **(B)**. Open columns, cortex; solid columns, hippocampus. Data are expressed as means ± SD. Analysis was performed using confocal microscopy and counting the number of co-labeled cells on five sections per animal (*n* = 3 rats for GFP/NeuN and GFP/GFAP, *n* = 3).

We then characterized the cellular specificity of scAAV2/9-2YF-NF8-d1-EGFP-mediated transgene expression in KA-treated rats. In both the hippocampus and the cortex, the vast majority of GFP-positive cells were neurons, with a very small percentage of labeled astrocytes (**Figure 8**) and no oligodendrocyte or microglial cells (data not shown)

## DISCUSSION

Gene delivery to the whole brain has been described following intravenous (Duque et al., 2009; Foust et al., 2009; Mccarty et al., 2009; Fu et al., 2011; Gray et al., 2011) or intra- CSF rAAV administration (Fu et al., 2003, 2010; Samaranch et al., 2012, 2013; Bucher et al., 2013; Gray et al., 2013). Important differences exist in global efficiency between different regions of the brain after intracisternal vector injection, the superficial regions in the immediate vicinity to the cranial sub-arachnoid space being more efficiently transduced than the deeper regions (Samaranch et al., 2012). These data suggest that penetration of the viral particles into deep brain layers is limiting.

Here, we show that intracisternal injection of AAV2/9 leads to gene transfer into neurons, astrocytes, and oligodendrocytes with the highest efficiency in cortex, hippocampus, and cerebellum and a lower efficiency in the striatum, and substantia nigra. Additionally, we show that a rationally designed mutation replacing two surface tyrosine supposedly involved in the recognition by the ubiquitin-proteasome complex, by a phenylalanine residue (Dalkara et al., 2012) enhanced global gene transfer efficiency. Interestingly, the ratio of glial versus neuronal transgene expression was modified by the 2YF mutation in the hippocampus (but not in the cortex), consistently with previous data obtained after intravenous injection (Dalkara et al., 2012; see Supplementary Figure 3).

Finally, we demonstrate that in response to systemic KA administration, transgene expression from a NFκB-responsive promoter was induced in the hippocampus and the cortex but not in the cerebellum. Our data are consistent with a previously published study showing that the expression of NFκB was increased in the cortex and slightly decreased in the cerebellum following intraperitoneal injections of KA (Rong and Baudry, 1996).

**FIGURE 9:**
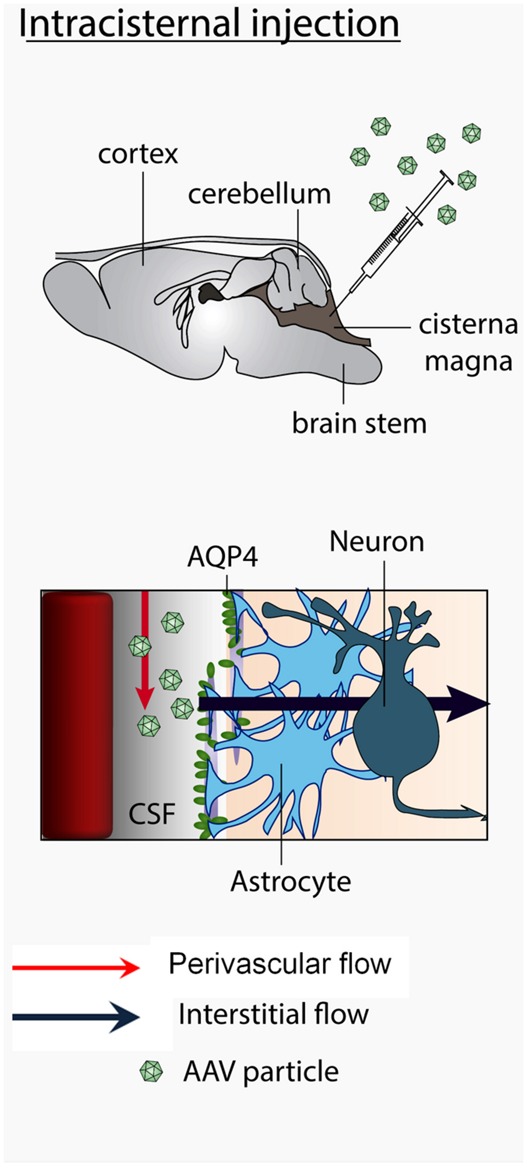
**Mechanism of trans-CSF-brain barrier AAV2/9 viral particles entry into the brain.** Viral particles travel with the CSF in the subarachnoid space, enter the brain via the perivascular Virchow-Robin spaces and further flow in the brain interstitial fluid. The CSF influx is facilitated by aquaporin-4 expressed by perivascular astrocytes.

The CSF produced in the choroid plexus is thought to travel to the cisterna magna, then in the Virchow-Robin subarachnoid space and flow into brain parenchyma along cerebral arteries. Molecules dissolved in the CSF are exchanged between perivascular spaces and brain interstitial fluids from which they can penetrate the brain parenchyma through the spaces between astrocytic feet and the basal laminae. These exchanges were suggested to require aquaporin-4 (Iliff et al., 2012; see **Figure 9**). The brain penetration properties of molecules administered into the cisterna magna varies with the size of the molecules. Small dyes enter the brain by diffusion into the interstitial fluid through gaps between astrocytic feet and the capillaries basal laminae whereas large molecules (2000 kD) similar to AAV particles (MW of approximately 4000 KD) remain confined in the perivascular spaces. They do not enter the interstitial fluid (Iliff et al., 2012). From the cisterna magna, viral particles are thus expected to circulate in the Virchow-Robin subarachnoid space and enter the brain along spaces around penetrating blood vessels. The mechanism by which AAV particles, enter the brain interstitial fluid is unknown. The fact that AAV2 particles transduce the brain after intracisternal delivery (Fu et al., 2003) exclusively in the presence of mannitol, suggests that the up-regulation of expression of aquaporin 4 by mannitol (Mccarty et al., 2009; Fu et al., 2010) may play a role of the entry of particles (Arima et al., 2003). In contrast, cisternally injected AAV2/9 particles enter the brain regardless of the presence of mannitol or sorbitol. However, the use of sorbitol (Gray et al., 2011) or mannitol (Bockstael et al., unpublished observations) enhanced transduction efficiency. Mannitol also enhanced *trans*-brain–blood barrier gene delivery mediated by AAV2/9 (Fu et al., 2011). Altogether these data suggest that the mechanism of viral particles entry into the brain parenchyma after intravascular and intra-CSF administration might share at least a common pathway involving the aquaporin 4 water channel.

Compared to intravenous injections, injections into the CSF were shown to reduce the bio-distribution of vectors into peripheral organs (Gray et al., 2013) as well as avoiding the inhibitory effect on gene transfer mediated by anti-AAV neutralizing antibodies (Gray et al., 2013). However, a strong inflammatory response to non-self-transgene products and elimination of transduction was observed in primates (Samaranch et al., 2014). This can be explained by the presence of activated T secreting cytokines at the choroid plexus-brain interface which can in some circumstances enter the CNS (Schwartz and Baruch, 2014).

We have previously established a proof-of-concept for disease-inducible gene delivery in the brain using an AAV vector responding to NFκB activation as a probe for inflammatory responses (Chtarto et al., 2013). In this study, the reporter vector was injected focally in the parenchyma prior to a systemic KA injection and mediated inducible and specific transgene expression in the hippocampus but not in the cerebellum, regions, respectively, known to be affected or spared by the pathology (Rong and Baudry, 1996). However, such a focal approach relies on the knowledge of the mechanism of disease induction/progression in the animal model.

Vector delivery in the CSF allows to reveal the pathology as well as to deliver therapeutic factors in several affected brain regions after a single vector injection and in a disease-regulated manner. However, in order to reflect the regional specificity of the disease, the distribution of the transgene product should be homogenous throughout the brain. In our study, the distribution of transgene expression is heterogeneous, with a higher efficiency in cerebellum, cortex, and hippocampus and a limited efficiency in deeper regions such as striatum. Methods to stimulate the perivascular pump or increase the functionality of water channels at the astrocytic feet could enhance viral particle penetration into the brain.

In other recent studies, the model of CSF circulation has been challenged and it has proposed that CSF is reabsorbed into brain capillaries (Pardridge, 2011). Thus AAV2/9 viral particles might first enter the brain capillaries than subsequently enter into the brain parenchyma through the blood-brain-barrier.

## AUTHOR CONTRIBUTIONS

Conceived and designed the experiments: Abdelwahed Chtarto, Liliane Tenenbaum, Olivier De Witte, Marc Levivier. Performed the experiments: Olivier Bockstael, Abdelwahed Chtarto, Catherine Melas. Analyzed the data: Olivier Bockstael, Abdelwahed Chtarto. Contributed reagents/materials/analysis tools: Olivier Bockstael, Abdelwahed Chtarto, Deniz Dalkara, Liliane Tenenbaum. Wrote the paper: Abdelwahed Chtarto, Liliane Tenenbaum, Olivier Bockstael.

## Conflict of Interest Statement

The authors declare that the research was conducted in the absence of any commercial or financial relationships that could be construed as a potential conflict of interest.
